# Monopolistic Control of Medical Literature

**Published:** 1897-04

**Authors:** 


### MONOPOLISTIC CONTROL OF MEDICAL LITERATURE.

LETTER FROM DR. GEORGE M. GOULD.

PHILADELPHIA, Pa., March 4, 1897.

*To the Editor :*

SIR—The editors of the *American Year-Book* must be gratefully sensible of the courtesy and general justice of your remarks in reviewing their work in the March issue of your valuable journal, and especially as regards the wisdom of the physician taking a goodly number of medical journals and the impossibility of any digest replacing his subscriptions to these serials. I was astonshed and pained, however, by your remark that the profession has no interest in the monopolistic control of medical literature.[1] Is it not plainly to the interest of every individual contributor, and especially when he gives his contributions without any compensation, to have it abstracted and brought before his colleagues by digests and reviews ?

You certainly, also, cannot be unaware of the great advantage to medical science and progress generally of the German *Jahresberichte*, *Jahrbücher*, and the like. Is it not most evident that these epitomisations of knowledge are of the profoundest practical advantage in bringing before every member of the profession the elements of progress and the scattered advances made all over the world ? How, then, can it be said that an attempt to prevent this dissemination on the part of a lay publisher, carried out persistently in the case of the only two thoroughgoing attempts that have been made in English (*Sajous's Annual* and the *American Year-Book*), has no professional interest ? Surely, Mr. Editor, you had not

1. What the JOURNAL said was, in effect, that it had no special interest in the differences between Dr. Gould and Wm. Wood & Co. We have kept the matter out of our columns until now ; but having been requested by both sides to let them speak, we reluctantly consent.—EDITOR.

considered the matter carefully. The two following letters make it plain that it is a matter of commercial policy on the part of Wood & Co., and the same policy carried out for eight years in reference to Dr. Sajous's excellent annual proves that the profession must see to it that its gratuitously-given contributions shall not be used by a purely commercial firm to do injury to individual medical men, by lessening the circulation of their discoveries and clinical results ; the profession should not be prevented from supplying every brother of the guild with the scattered results of progress gained. That no other publisher in the world has taken such a stand should arouse the profession to the danger that would ensue from a possible like action of all other publishers. Will you not reconsider your editorial position on this important and by no means personal or special policy ? These letters, in conjunction with the editorial statement in the *Medical Record* of February 13, 1897, make it clear what motives are at work :

(Copy.) NEW YORK, September 10, 1896.

*Dr. Geo. M. Gould :*

DEAR SIR—Replying to yours of the 7th inst. respecting the *Year-Book of American Medicine and Surgery*, we would be very pleased to do anything we could to accommodate you personally, but, very candidly, we do not feel disposed to do anything whatever to assist your publisher. His methods of work with authors and booksellers are such as do not commend him to us as a competitor in business. We say this without a particle of animosity, simply in a cold-blooded judicial way. We are not willing to assist anyone to eat into our business, if we can avoid it. Kindly consider this statement of facts to be entirely aside from our cordial good-will towards yourself.

Very respectfully yours, WM. WOOD & CO.

---

(Copy.) OCTOBER 27, 1896.

*Dr. Geo. M. Gould :*

DEAR SIR—In reply to yours of yesterday with reference to selling casts of illustrations to Mr. Saunders, we beg to say that we have never considered the reprinting of articles from our journals to be of any appreciable value to us or to others. Considering the fact that there are already more medical books published than profitable sale can be found for, we have always thought books of that kind an injury to the medicine book-publishing business. Mr. Saunders has called upon us relative to this matter and stated the case fully and courteously. As it is strictly a matter of business, we would very much prefer that it should be left for settlement with the business parties interested.

Very respectfully yours, WM. WOOD & CO.

By GEO. S. NILES.

Whether this is "strictly a matter of business" in which professional interests are not concerned is a question for the profession to ponder most seriously before deciding. With the quarrels of lay publishers we have no concern, so long as the dissemination of medical literature is not hindered; but when a lay publisher attempts to monopolise that literature after its gratuitous gift to him by a profession above all things averse to scientific monopoly, it is time that the professional conscience and interest were aroused.

Cordially yours, GEO. M. GOULD.

119 S. 17th Street.

---

REPLY OF MESSRS. WILLIAM WOOD & CO.

NEW YORK, March 11, 1897.

*To the Editor:*

SIR—Referring to the foregoing communication of Dr. Gould we beg to state that for nearly two years Mr. W. B. Saunders, the publisher of the work of which Dr. Gould is editor, has been most frequent in his requests for illustrations from our publications. While it is not unusual, in the courtesy of the trade, for one publisher to extend this accommodation to another, yet among the older houses it is a favor asked with moderation, if not with hesitation. Mr. Saunders, a new-comer in the ranks of medical publishers, has, on the contrary, apparently felt no such delicacy, but in the past eighteen months has applied to us for the cuts, or the permission to use them, of nearly seventy illustrations. We naturally became somewhat impatient, not only of this wholesale use of our facilities, but by the trouble and annoyance attending the unprofitable satisfying of his request, and told Mr. Saunders so.

Last September, Dr. Gould, for some reason, injected himself into this business correspondence and wrote us, asking, in a general way, if he might have some cuts from our journals to illustrate abstracts therefrom. We replied that, while personally most friendly disposed toward him, we did not much care to assist his publisher. Dr. Gould then wrote us, October 26, 1896, criticising our views in the matter, and requesting a reconsideration. To this we replied that our views were unchanged, that we had fully stated them to Mr. Saunders, who had meanwhile called upon us, and requesting Dr. Gould to permit his publisher and ourselves to settle this business transaction.

On November 2d, in response to his request, we made for Mr. Saunders a set of electrotypes of some cuts which he desired. Notwithstanding this, Dr. Gould again wrote us the remarkable letter, (dated December 1, 1896,) a copy of which is as follows:

"However much I may dislike correspondence with you, and despite your absurd contention that I, as editor of the *American Year-Book*, have nothing to do with the source, choice or illustration of articles used in our report, I reluctantly write you once more in reference to the matter. I wish to learn definitely if you still persist in refusing my request of permission to abstract, quote and reproduce the illustrations, at my pleasure and that of the departmental editors, from articles published in your journals? I ask the question again, because, after your repeated refusal, and when, after great expense and trouble, the abstracts of such articles had finally been cut out of the make-up, when, therefore, the illustrations were no longer of any earthly use to us, you tumble in upon us a lot of electros which, if sent when requested months ago, would have been gladly used. This sort of method of doing business is wasteful, silly and disgusting. I must at once notify my departmental editors to use no material from your journals during the coming year if you persist in your refusal. There must be a definite understanding. After a year or more of correspondence everything is higgledepiggledy, which must be ended. I must have a categorical answer to the following questions:

"*Do you or do you not give me and my editors the right to abstract, quote and reproduce illustrations from your journals at our pleasure?* Please answer this simply *yes* or *no*. If in three days I receive a negative answer, or no answer at all, I shall instruct the editors accordingly—and then, I may add, I shall at once proceed to let the medical profession know the stand you have taken.

"(Signed) GEORGE M. GOULD."

Such a communication we consider unworthy of notice and as placing the writer below the plane of our further consideration. We have since ignored him.

We never refused any request of Mr. Saunders to abstract from our publications, nor, with one exception a long time ago, have we ever declined to allow him the use of cuts.

Dr. Gould's statements in his letter to the members of the medical profession, of December, 1896, which has been published in several journals, are, therefore, not only unjust and misleading, but untrue.

Had the whole matter been left entirely to Mr. Saunders and ourselves we have no doubt that all parties would finally have been satisfied. Dr. Gould, by his interference, has forced an issue not contemplated by us by his letter of December 1, 1896, by his subsequent letters to the medical press and by his last abusive letter to Dr. Shrady, which, as published in the *Journal of the American Medical Association* of March 6, 1897, is as follows:

MONOPOLISTIC CONTROL OF MEDICAL LITERATURE.

PHILADELPHIA, February 20, 1897.

*To the Editor of the Medical Record:*

SIR—In your editorial columns (issue of February 13, 1897, pp. 234-5,) you permit a letter to be published as your own ("Both editors and publishers") which is so diametrically opposite in view to that you personally gave myself (in company with Dr. Pyle) when calling upon you, that I must beg for a word of explanation in reply. Where is now your "dead line" across which you repeatedly said your publishers could not go in control of your editorial utterance? Moreover, who are the "editors" of the *Medical Record*? Your announcement cites yourself as the single editor. If there are others they should certainly be named, as the profession should know its members who take this attitude to professional interests.

And now, sir, of what use is it for you to allow such statements to appear in your columns as the following?—that you would be "the last to countenance any action which might interfere in the slightest degree with the fullest and freest dissemination of medical knowledge." "The *Medical Record* has always allowed the free use of the original matter contributed to it for all reputable purposes, provided that due credit is always given to the original source of publication," and the like. Now, my dear doctor, you know this is all untrue, and that it is easily proved untrue.

Let me first attend to the insult conveyed in the words, "reputable purposes." Of course, you were fully aware of the intended inference and insult. Since your employer's refusal to allow the *American Year-Book* to use articles from your columns in its digests which, you say is never refused for "reputable purposes," it follows plainly enough that all the editors of the *American Year-Book* are, in your opinion, engaged in a disreputable or nonreputable undertaking. We thank you for your high opinion of us, but can hardly agree with you. Neither does the medical profession, judging from appearances.

Let me make another quotation from your editorial: "The letter above referred to, while plausible on its face, is misleading in its intent, inasmuch as it implies that our course in this one instance might be considered as characteristic of our general policy, than which nothing is more untrue." This aspect of the affair is evidently worrying your publisher a great deal and so he makes the most sweeping denials possible that he is not guilty of trying to prevent the dissemination of medical knowledge. The denial will avail nothing. American physicians have already decided that the *American Year-Book* is not disreputable, and so long as you and your publisher keep your reasons absolutely hidden you will be credited with a very evident and very sordid motive. Your contributors must smile derisively when they hear you say that you "would be the last to countenance any action," and the like, and yet in the same editorial you confess that they cannot have their articles noticed in the *Year-Book*. Something more than stentorian proclamations of self-righteousness is necessary to deafen them to such wonderful logic.

What, then, will they say when they learn that this one instance is by no means single, and that these same "publishers and editors" have for eight years persistently refused the same privileges to Dr. Sajous and his sixty-six editors of the *Annual*, a publication that has had to go on as best it could all these years with the same denial on the part of the *Medical Record* publishers. Of course, the *Annual*, again by necessary inference, is "disreputable," but, again also, the profession disagree.

Now, sir, as a physician you well know the wondrous service to the science, the art and the practice of medicine rendered in Europe by the epitomes of the world's medical knowledge, *Jahrbücher*, *Handbücher*, and the like. Their use is simply incalculable. Your publisher (and also you, since you pronounce his cause just,) must yet settle with an outraged profession for refusing to allow the use of the gratuitously given literature over which the profession has unwittingly given you a legal control, and in the only two "reputable" and thoroughgoing attempts to supply this want to English and American medical men.

And I cannot see that our just indignation is softened any by the claim that this outrageous action is motived now on "personal," now on "sound business" reasons. It seems to me that such a claim makes your case all the worse before a professional jury, and that in making such a sorry excuse you confess that you are willing to harm the profession to any degree so that you satisfy some private grudge or financial aim. This is all the more noteworthy coming from a firm that has for many years in similar handbooks made most sweeping and wholesale use of all serial medical literature, not only that given gratuitously to lay publishers by the profession, but whole books and by the dozens. (See Dr. Murrell's letter in *British Medical Journal*, October 11, 1890.)

I had intended not noticing the personal insult levied at me. Despite your publisher's and your own great solicitude to reduce this controversy to "personalities," "peculiarities" and "sound business reasons," neither of these things has anything whatever to do with the whole matter. If your reasons are honorable, for heaven's sake out with them ! "If not a settled policy," we may be able to find more than nine years of history and two competing publications to prove it. I by no means intend that the principles at issue shall be allowed to be made confused by any such alarums to distract the enemy's attention. In the present instance, your contemptible charge that I have no higher motive in this whole matter than to advertise a book of which I am one of twenty-eight editors, and that I allow my publisher to use my editorial utterances purely as his advertising medium, is—well ! What may a gentleman say to such an insult ? I am perfectly willing to leave such impertinence to be answered by the three publishers with whom I have had close business relations and with the readers of the *Medical News*. Some men, my dear sir, are very prone to ascribe low motives to others, but the reason is proverbially known. Oh, no, the instances are too few, but they exist, of editors who are not the jumping-jacks of their publishers. No publisher could have forced into my editorial columns such an editorial as this of yours under discussion. Mr. Saunders had no hand in the writing of that circular, was not consulted about it, knew nothing about when or where it was mimeographed, mailed, and the like. It was solely and wholly my own work. He has no more knowledge concerning this letter. Instead of all this shameless trying to turn the issue and of all these blank denials, it would be far more to the point if you would explain to your contributors how it is not against their interests to refuse to allow their gratuitously given articles to be abstracted and illustrated in the only two (not in only one, as you say,) earnest attempts that have been made to get up in English something comparable to the immensely serviceable and necessary German *Jahrbücher* and epitomes of medical progress. Also please explain how it is your action does not prevent the dissemination of medical literature. If it does not do so, the necessary inference is that your two journals, in your own estimation, contain nothing worthy of being thus used in a résumé of medical science and progress. Lastly, will you not explain to the sixty-six departmental editors of *Dr. Sajous's Annual* and to the twenty-seven gentlemen connected with the *American Year-Book* in what way these two publications are disreputable or nonreputable ? Are they not quite as reputable as your publisher's similar Handbook, and the like, and his pirated whole books of our English colleagues republished without their consent, and sometimes against their command and to their financial loss, as in those instances when other American publishers would have paid them for the right to reprint had it been possible to guard against the American pirate ?

I reserve a little clarifying discussion of your publisher's position until a later date.

Cordially yours, GEO. M. GOULD.

119 S. 17th Street.

The original of this was sent to Dr. Shrady, including some additional personalities, cut out in the published copy, with a request that it be published in the *Medical Record.*

We regret extremely that a man of Dr. Gould's previous reputation should have made such an exhibition of himself, and we think quite likely that he will endeavor to pursue this matter further if he can get any medical journal to print his letters. As we have said before, we shall not enter into any controversy with Dr. Gould, but we must emphatically deny his, we believe, intentionally misleading statements ; and if it be a question of veracity between Dr. Gould and the firm of William Wood & Co., we are willing to let it rest on that basis.

Thanking you for your courtesy, believe us, as ever,

Very truly yours, WM. WOOD & CO.

